# Single‐Cell Mobility Analysis of Metastatic Breast Cancer Cells

**DOI:** 10.1002/advs.201801158

**Published:** 2018-10-31

**Authors:** Jialang Zhuang, Yongjian Wu, Liang Chen, Siping Liang, Minhao Wu, Ledu Zhou, Chunhai Fan, Yuanqing Zhang

**Affiliations:** ^1^ School of Pharmaceutical Sciences Sun Yat‐sen University Guangzhou 510006 P. R. China; ^2^ Department of Immunology Zhongshan School of Medicine Sun Yat‐sen University 74 Zhongshan 2nd Road Guangzhou 510080 P. R. China; ^3^ Department of General Surgery Xiangya Hospital Central South University Changsha Hunan 410008 P. R. China; ^4^ Laboratory of Physical Biology Shanghai Institute of Applied Physics Chinese Academy of Sciences Shanghai 201800 P. R. China

**Keywords:** cell migration, MCPIP1, metastasis, microfluidic, single‐cell anlalysis

## Abstract

Efforts have been taken to enhance the study of single‐cells, however, the task remains challenging because most previous investigations cannot exclude the interactions between single cells or separately retrieved cells with specificity for further analyses. Here, a single‐cell mobility analysis platform (SCM‐Chip) is developed that can not only real‐time monitor single‐cell migration in independent niches but can also selectively recover target cells one by one. The design of each channel with a single‐cell capture unit and an outlet enables the system to place single cells in different isolated niches with fluidic capture and to respectively collect target cells based on mobilities. SCM‐Chip characterization of breast cancer cells reveals the presence of high‐ and low‐migratory populations. Whole‐cell transcriptome analysis establishes that monocyte chemotactic protein induced protein 1 (MCPIP1) is related with cell mobility; cells with a high expression of MCPIP1 exhibit low mobility in vitro and metastasis in vivo. The SCM platform provides a generic tool for accurate single‐cell isolation and differentiation that can be readily adapted for the study of cancer and drug development.

Cancer metastasis represents a serious problem in cancer therapy, as 90% of cancer‐associated mortality is due to metastatic recurrence.^1^ Collective migration plays a crucial role in cancer invasion and metastasis;^2^ however, it provides no information about the migratory mechanisms of single cells. Evidence is mounting that metastatic cells exhibit increased genetic instability when compared with nonmetastatic cells, and such heterogeneity results in not only a difference in cell movement but also diversity in the expression of multiple genes.^3^, ^4^, ^5^ The study of cell migration at the single‐cell level is therefore important for the understanding of this complex process and heterogeneous biological system. Currently, a small number of existing advances have introduced novel methods to study single‐cell migration.^6^, ^7^, ^8^, ^9^, ^10^, ^11^, ^12^, ^13^, ^14^ However, most of these methods seem to be performed as multiple single‐cell arrays instead of applying a real single‐cell microenvironment. Since dispersive cells still share the same niche within these platforms, previous studies selectively ignore the interactions between different individual cells, such as secreted cytokines, which can influence cell behavior at the single‐cell level.^15^, ^16^ Microfluidic approaches have been currently employed to isolate single cells based on cell surface markers or functional characteristics, but these methods were restricted to collect a subpopulation of single cells rather than true specific single cell one by one.^17^, ^18^, ^19^, ^20^ Undoubtedly, precise single‐cell retrieval is still highly challenging for single‐cell study. However, novel cell detachment with specificity, such as photomechanical and hydrodynamic single‐cell release, needs complicated fabrication process, additional optical approach, and careful fluid control.^21^, ^22^, ^23^ Thus, we designed and fabricated a novel microfluidic platform that has the ability to maintain a relatively independent microenvironment for a single‐cell mobility array and to readily retrieve target cells for further analyses.

Our single‐cell mobility chip (SCM‐Chip) allows: 1) efficiently capture single cells, 2) technically maintain independent niche for single‐cell study, 3) easily recover specific cells separately, 4) sufficiently provide enough single cell sample for Single‐cell RNA‐seq. Since all the microchannels and outlets within SCM‐Chip were separate units, single cells were kept segregated on a limited area during the whole experiment and could be collected individually. After single‐cell mobility array, a small number of breast cancer cells with different migratory capacities were retrieved from the SCM‐Chip, followed by single‐cell RNA sequencing for high‐migratory cells versus low‐migratory cells, which indicated a series of differentially expressed genes as potential regulators of cell mobility. Among these candidate genes, monocyte chemotactic protein induced protein 1 (MCPIP1), which is highly expressed in low‐migratory cells, was identified as a novel suppressor of cell mobility in breast cancer. Increasing MCPIP1 can not only reduce cell migration but also inhibit lung metastases in vivo. Moreover, cross‐talk exists among the MCPIP1 and transforming growth factor‐β (TGF‐β) pathways in breast tumours. The finding of MCPIP1 in breast cancer progression suggests that our approach was able to measure true biological variation in a population of breast cancer cells at the single‐cell level.

The SCM‐Chip is a multichannel microfluidic chip resembling a clover; it was assembled by bonding a polydimethylsiloxane (PDMS) layer with a microfluidic pattern to a round glass slide (Figure S1, Supporting Information). The device (**Figure** **1**a,b) consists of a main inlet (Region 1), 3 branched networks of channels that extend into three arrays of 32 parallel lanes (Region 2), and 96 separated outlets (Region 3). Each channel contains one ultraminiaturized hook with the open end pointing toward the inlet, which is placed at the middle of the channel. As the opening of the hook (14 µm) and the gap of the hook (3 µm) are much smaller than the bypass channel (20 µm), it can sterically capture a cell (Figure S2, Supporting Information). Previous studies of this microstructure have demonstrated a high capture yield of single cells.^24^, ^25^, ^26^ The long microchannel was designed to extend a distance of 1000 µm from the hook to both ends, which can not only provide enough space for single cell movement but also retain all signalling molecules within the migration lane. By maintaining this isolated niche for single cell, interfere of local microenvironment and cell–cell interaction were excluded while only gene variance should be account for the difference of results of cell mobility. Since all the channels were quite separate and each channel has its own outlet, single‐cell migration was supposed to be independent from each other under static condition. Moreover, this design was also essential for precise single‐cell retrieval because only one cell in principle can be obtained from one outlet. To gain migration profiles and enrich cells of interest, a standard procedure was developed (Figure 1c,d): 1) A particular amount of cell suspension is loaded into the device through the inlet flow, and then, the divided cell suspension flow reaches the microscale hook (Video S1, Supporting Information). A single cell is captured by the single hook, and the others flow along the bypass channel. 2) Cell‐free medium is quickly loaded to wash out the uncaptured cells within the microchannel to the outlets by continuous fluid flow. 3) All channels of the SCM‐Chip are photographed by a microscope after 18 h of migration, and the images are subjected to accession of the migration profiles (Figure S3, Supporting Information). 4) Functional enrichment is carried out to selectively retrieve those cells with various mobilities from different outlets by flowing trypsin into the main inlet and carefully collecting with pipette tips (Video S2, Supporting Information). During the experiment, both the cell population and the flow rate were investigated to determine the optimum conditions for single‐cell capture. Finally, the optimal cell density was determined to be 5 × 10^6^ per mL. Similar single‐cell capture yields could be observed among different flow rates, and thus, the lowest flow rate (2.5 µL min^−1^) was chosen. Three breast cancer cell lines were chosen to challenge the effectiveness of the single‐cell capture, and high cell capture rates (>80%) have been achieved for these cells (Figure S4, Supporting Information). After single‐cell capture on the chip, low‐serum culture medium (1%) was introduced into the device, allowing the cells to maintain their biological function during the migration process. Since all of the cells are trapped in the hooks within the microchannel while all hooks are at the same horizontal level, all of the “runners” should be placed on a common start line (Figure S5, Supporting Information). The long channel in the SCM‐Chip allows single‐cell migration without interactions while cells are exposed to individual niches. Thus, the migration distance can be easily attained by real‐time monitoring of the final cell location during the entire experiment. Finally, specific cell retrieval can be achieved based on the mobilities of single cells.

**Figure 1:**
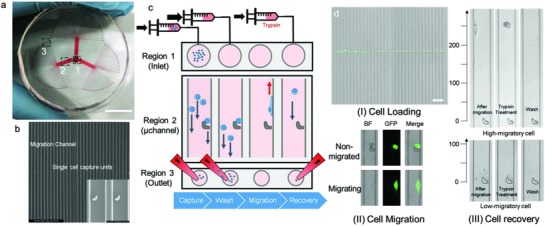
Working principle of the SCM‐Chip. a) Photograph of SCM‐Chip indicating the inlet. Scale bar: 15 mm (Region 1), one of the three multi‐microchannel branches (Region 2), and different isolated outlets (Region 3). b) SEM of the chip with higher magnification of microchannel and magnified hooks for single‐cell capture. Scale bars: 500 and 50 µm. c) Work flow for single‐cell mobility array using SCM‐Chip, including single‐cell capture, cell washing, single‐cell migration, and specific cell recovery. d) Characterization of MDA‐MB‐231 cell loading (I), scale bar, 200 µm, single‐cell migration (II), and functional retrieval on‐chip (III).

First, the SCM‐Chip successfully demonstrates its capacity for single‐cell mobility profiling of three breast cancer cell lines with distinct migratory characteristics. Previous studies have revealed that MCF‐7 cells exhibit less metastatic ability than MDA‐MB‐231 and SUM‐159 cells.^27^, ^28^ In this study, metastatic MDA‐MB‐231 and SUM‐159 cells showed stronger migratory (**Figure** **2**a,b; Figures S6–S8, Supporting Information) than nonmetastatic MCF‐7 cells. We performed cumulative distribution analysis of the migration distance and found that the results were correlated with the wound‐healing assay and transwell migration assay, which suggested the reliability of our single‐cell mobility detection system (Figure S9, Supporting Information). The results of this study were also consistent with previous collective migration reports of breast cancer cells^29^, ^30^ (Tables S1 and S2, Supporting Information), which reported that MDA‐MB‐231 cells exhibit a cell speed range from 0.10 to 0.80 µm min^−1^, while the velocity of MCF‐7 cells was 0.16 µm min^−1^. Moreover, the histograms of cell migration distance among three breast cancer cell lines suggest that the distribution of cell mobility should be left skewed (Figure S10, Supporting Information). We reasoned that the non‐normal distribution of the migration distance distribution could origin from cell heterogeneity. In our previous study, MCF‐7, which was globally identified as low‐migratory cells, exhibited a migration distance below 100 µm after 18 h while MDA‐MB‐231, which was identified as high‐migratory cells, tend to reach more than 150 µm after 18 h movement. Moreover, recent on‐chip studies^6^, ^7^, ^8^, ^9^ also suggest that MCF‐7 cells shows a locomotion of 0.05 to 0.16 µm min^−1^ (54–172 µm per 18 h). Based on these findings, we defined a cell which can reach greater than 100 µm after 18 h (0.09 µm min^−1^) as high‐mobility cells, while for low‐mobility cells, the opposite is true. Meanwhile, no difference between up‐migration and down‐migration among the three cell lines, such as the migration distance and the amount of migrated cells, may reflect that cell migration on the chip seems to be a random movement (Figure S11, Supporting Information). The results of real‐time monitoring of the SCM‐Chip indicated that majority of migrating cells (93.1%) were tend to attach on left or right side wall and travel toward the inlets or outlets in SCM‐Chip (Figure S12 and Video S3, Supporting Information). In this study, cells confined in narrow (w × h × l, 30 × 15 × 1000 µm^3^) channels preferentially walk along the microchannel leading to a persistent cell migration in the same direction. This phenomenon can be termed “contact guidance,”^31^ the 3D topographies of SCM‐Chip microchannel increase the occurrence of straight cell trajectory. These results indicated that the SCM‐Chip enables us to evaluate single‐cell migration behavior, and cells with different levels of metastatic ability exhibited different mobilities on the chip.

**Figure 2:**
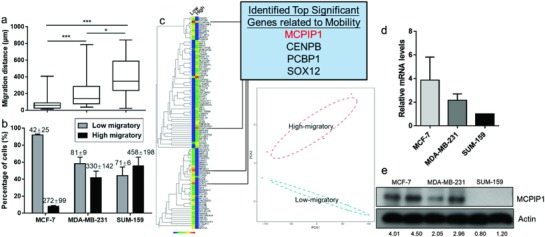
Increased MCPIP1 expression among low‐migratory cells. a) Comparison of the migration distances and percentage of high‐migratory versus low‐migratory cells (mean ± SD is shown in corresponding bar plots to represent the results) b) for MCF‐7, MDA‐MB‐231, and SUM‐159 cells. c) Left: Comprehensive heat map showing the expression levels of genes between high‐migratory and low‐migratory cells. Right: Row corresponding to the top 265 different expressed genes across these two subpopulations (upper panel). Principal‐component analysis (PCA) of the transcriptome of high‐migratory and low‐migratory cells sorted by SCM‐Chip from MDA‐MB‐231. Cells from the same group are shown as symbols of the same color. PCA1 and PCA2 represent the top two dimensions of the genes showing all expression among cells with different mobility, which accounts for 55.1% and 18.1% (lower panel). d) qRT‐PCR analysis of MCPIP1 mRNA level in MCF‐7, MDA‐MB‐231, and SUM‐159 cells. e) Western‐blot analysis of MCPIP1 protein level in MCF‐7, MDA‐MB‐231, and SUM‐159 cells.

Obvious heterogeneity of the migration of single cells in the device was observed among these cells, which raises the question of what drives some cells to move faster than others. We sought to answer this question by determining the differences in gene expression between high‐migratory and low‐migratory cells. A small number of MDA‐MB‐231 cells with different mobilities sorted in the SCM‐Chip were subjected to single‐cell RNA‐seq using the Smart‐Seq2 method.^32^, ^33^ To further dissect the two groups with different mobility sorted by SCM‐Chip, principal component analysis (PCA) was applied, which confirmed the difference between high‐migratory groups and low‐migratory groups. Moreover, the relative long distance within these two groups of cells in the PCA analysis indicated cellular heterogeneity (Figure 2c). The results suggest that cells with high mobility were enriched in genes related to DNA binding, cell adhesion, locomotion, and cellular component organization (Figure S13, Supporting Information). Gene set enrichment analysis indicated that most of the top‐ranked sets have associations with cellular process, metabolic processes, and biological regulation (Figure S14, Supporting Information). Among these candidate genes, we focused on MCPIP1, a defined gene up‐regulated by approximately eightfold in low‐migratory cells (*p* < 0.001 and false discovery rate (FDR) < 0.01) (Table S3, Supporting Information). Since MCPIP1 is well known as a ribonuclease, few studies have linked MCPIP1 to cancer metastasis.^34^, ^35^, ^36^ Our unpublished data suggested that the expression of MCPIP1 was related to the microcluster formation of tumour cell, which might be account for the regulation of cell mobility. Moreover, a recent study indicated a concordance between decreased levels of MCPIP1 and worse survival,^37^ which also supports that MCPIP1 could be a potential new regulator of metastasis in breast cancer. Together, these findings indicate that MCPIP1 could be responsible for cell mobility.

A further study of gene expression among MDA‐MB‐231, SUM‐159 cells, and MCF‐7 cells illustrated the association between MCPIP1 and cell mobility. We noticed the asymmetric distribution of the gene expression of MCPIP1 among these three cell lines (Figure 2d,e), which is associated with the cell mobility. For example, the cells with relatively high MCPIP1 expression (MCF‐7) seem to reach a less migration distance than those cells with lower MCPIP1 expression (SUM‐159 and MDA‐MB‐231). Interestingly, for the highly metastatic MDA‐MB‐231 and SUM‐159 cancer cells, MCPIP1 was expressed to a low degree or as undetectable at the protein level; however, MCPIP1 protein was highly expressed by the nonmetastatic MCF‐7 cells. These results confirm that breast cancer cells with different levels of MCPIP1 expression exhibit different migration profiles, which supports the particular relationship between MCPIP1 expression and cell mobility.

To investigate the biological effect of MCPIP1‐mediated cell migration, we determined the cell mobility of MDA‐MB‐231 cells after the transient transfection of MCPIP1 or a control vector (**Figure** **3**a,b). Compared with cells exhibiting a high‐expression of MCPIP1, cells with a low expression of MCPIP1 demonstrated an increased migration capacity under the same conditions (Figure 3c,d; Figures S15 and S16 and Table S4, Supporting Information). MCPIP1 transfection was found to prolong the formation of attachment and pseudopodia of migrating cells (Figure S17, Supporting Information). Thus, the delay in the change of cell morphology during the migration process in MDA‐MB‐231/MCPIP1 cells may be explained by specific functional genes.

**Figure 3:**
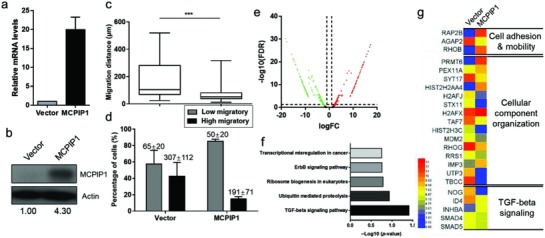
MCPIP1 reduces single‐cell migration and suppresses TGF‐β signaling. a) qRT‐PCR analysis of MCPIP1 mRNA level in MDA‐MB‐231/Vector and MDA‐MB‐231/MCPIP1 cells. b) Western‐blot analysis of MCPIP1 protein level in MDA‐MB‐231/Vector and MDA‐MB‐231/MCPIP1 cells. Comparison of the migration distances. c) Percentage of high‐migratory versus low‐migratory cells (mean ± SD is shown in corresponding bar plots to represent the results). d) For MDA‐MB‐231/Vector and MDA‐MB‐231/MCPIP1 cells. e) Volcano plot of differentially expressed gene between MDA‐MB‐231/Vector and MDA‐MB‐231/MCPIP1 cells. The red dots represent up‐regulation genes and the green dots represent down‐regulation genes. f) Enriched KEGG pathways in gene correlation among MCPIP1 overexpression cells. g) Heat map for mRNA related to cell migration and TGF‐β signaling between MDA‐MB‐231/Vector and MDA‐MB‐231/MCPIP1 cells.

To determine how MCPIP1 affects cell mobility, we conducted a whole‐transcriptome analysis using MCPIP1‐transfected MDA‐MB‐231 cells. Based on the finding of bulk RNA sequencing, we successfully identified potential genes that are involved in cells with a high level of MCPIP1. Gene ontology analysis was performed to determine correlations with biological function, cellular components, and molecular function (Figure S18, Supporting Information) among these genes. The whole‐transcriptome data were classified by accessing up‐regulated and down‐regulated genes between cells with different expressions of MCPIP1 (Figure 3e; Figure S19, Supporting Information). Further Kyoto Encyclopedia of Genes and Genomes (KEGG) analyses (Figure 3f,g) of our gene expression profile to a top‐ranked TGF‐β response signature indicated a global enrichment of TGF‐β‐suppressed genes in the presence of MCPIP1. High MCPIP1 expression levels in MDA‐MB‐231 cells led to the dysregulated expression of genes encoding key members of the TGF‐β pathway including downregulation of the ID4 and Noggin (NOG) and upregulation of the TGF‐β signalling inhibitor genes INHBA, which have been implicated in maintaining cell adhesion and migration.^38^, ^39^, ^40^ Thus, we speculated that the suppression of cell mobility may be dependent on the gene expression of MCPIP1 due to an alteration in TGF‐β signalling.

As the cell migration was attenuated by both the overexpression of the MCPIP1 gene and TGF‐β pathway inhibition, we investigated whether the inhibitory effect of MCPIP1 on cell migration is associated with the blocking of endogenous TGF‐β signalling. First, we assessed the single cell migratory profiles between breast cancer cells with different expressions of MCPIP1 (**Figure** **4**a,b; Figures S20 and S21 and Table S5, Supporting Information). As expected, cells with high MCPIP1 expression (MCF‐7/Vector and MDA‐MB‐231/MCPIP1) showed lower single breast cancer cell migration on the chip than cells with low MCPIP1 expression (MDA‐MB‐231/Vector). However, the inhibition of the TGF‐β pathway restored the levels of low‐migratory cells back to the levels seen with cells having high MCPIP1 expression. This result was perhaps not surprising, as the wound healing assay showed similar results that cells with low MCPIP1 expression exhibited low mobility, which was responsible for TGF‐β signalling inhibition (Figure S22, Supporting Information). Furthermore, properties of metastasis were then evaluated in a xenograft model. Mice challenged with MDA‐MB‐231/Vector cells, which were identified as low MCPIP1 expression cells, showed increased lung weight and a higher number of tumour nodules compared to those mice receiving cells with high MCPIP1 expression (Figure 4c–f). Our observations were consistent with the results that the addition of MCPIP1 expression suppressed cell migration in vitro (Figure 3c,d). In addition, upregulation of MCPIP1 has little impact on cell proliferation, which suggested the cancer cell survival should not be affected by MCPIP1 (Figure S23, Supporting Information). However, we cannot exclude the possibility of MCPIP1 might regulate the plasticity of tumour cells by the adaption of lung microenvironment, thus further study should be needed to evaluate. Meanwhile, haematoxylin and eosin staining confirmed that TGF‐β signalling inhibition resulted in the decreased metastatic ability of cells with low MCPIP1 expression in vivo. The results revealed that the inhibition of TGF‐β signalling could rescue the enhancement of decreased MCPIP1 expression on tumour metastasis and inhibit breast cancer invasion. Further study suggests that TGF‐β inhibition by SB431542 led to the increase of MCPIP1, which also confirms the impact of TGF‐β pathway on MCPIP1 (Figure S24, Supporting Information). However, total TGF‐β protein level exhibited no difference in cultured MDA‐MB‐231 cells with different expression of MCPIP1. We reasoned that MCPIP1 might be contributed to the regulation of mRNA stability of TGF‐β signalling instead of the protein expression because of its post‐translational activity. Taken together, these results indicated that the inhibitory effect on cell migration and metastasis of MCPIP1 might be associated with the suppression of TGF‐β signalling. Nevertheless, the mechanisms that mediate MCPIP1 in highly metastatic cancer remain unknown and need further investigation.

**Figure 4:**
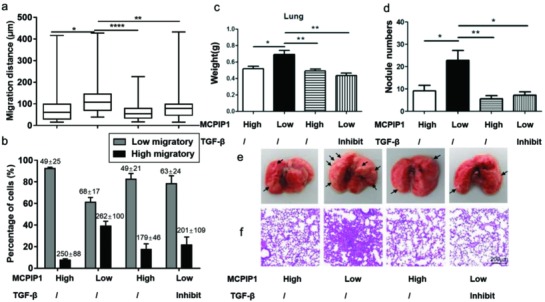
Inhibition of the TGF‐β pathway restores the MCPIP1‐dependent cell migration. a) Comparison of the migration distances and percentage of high‐migratory versus low‐migratory cells (mean ± SD is shown in corresponding bar plots to represent the results) b) for MCF‐7/Vector, MDA‐MB‐231/Vector, MDA‐MB‐231/MCPIP1, and SB431542 (10 × 10^−6^ m) pretreated MDA‐MB‐231/Vector cells. c) Quantification of the lung weight and d) the number of nodules on lungs of BALB/c nude mice. Representative images of e) lungs and f) haematoxylin and eosin staining of lung sections from BALB/c nude mice, scale bars, 200 µm.

The system described here can be used to recover cells with distinct mobilities independently by testing the migration distance after 18 h of movement on the chip. In this study, an ideal experimental model for cell migration in vitro study was established, which can be used for study cells' instinctive movements by setting an isolated channel for each single cell. Moreover, this platform could also be used for the study of rare cells, especially circulating tumour cells (CTCs), because even an individual patient's CTCs can be highly heterogeneous.^41^, ^42^, ^43^ Such an approach enables the study of CTC intrinsic invasiveness that can serve as a benchmark for the detection of metastasis. Although only 96 hook structures were implemented in this device, which is sufficient for the current study, this microarray can be extended to a system with 384 hooks or more by setting a uniform space between hooks within different microchannels (Figure S25, Supporting Information). Techniques developed previously have efficiently achieved single cell capture and the accurate detection of cell mobility, but none of them have provided the ability for functional isolation separately as this study did. Moreover, this approach is so simple that each biological laboratory equipped with microscope, constructed PDMS chip and syringe pump can fill in the fundamental requisite to collect target cell one by one in principle. Meanwhile, the SCM‐Chip enabled the study of single cells without effects from the microenvironment, which allows further study of the internal mechanisms of cancer cells. The SCM‐Chip can also provide single cell chemotaxis by introducing chemoattractants for cell migration into the main inlet.

Here we show a basic study of discovering new mediator of cell migration, which suggests the reliability of SCM‐Chip of single‐cell study. Our study demonstrates the importance of post‐translational mechanisms on tumour metastasis, especially MCPIP1, which might be associated with the decrease of cell mobility. Our results indicate that overexpression of MCIPIP1 should be related with the reduction of the ability of cells to migrate in vitro and invasiveness in vivo, which could be reasoned by the regulation of cell mobility. Further studies indicate that TGF‐β signalling may be involved in this process. Similar with the inhibition of cancer cell metastasis by the disruption of TGF‐β signalling, MCPIP1 overexpression can also result in the attenuation of cell migration. In this study, the aggressive invasion of cells with low MCPIP1 expression in vivo was restored by TGF‐β signalling inhibition. However, the metastasis of SB431542‐treated cells was still higher than that of cells with high MCPIP1 expression. This result suggested that the mobility of MCPIP1 overexpressing cells is inhibited not only through a TGF‐β‐mediated pathway but also through other signalling pathway(s), and in fact, the bulk RNA‐seq data have indicated that other pathways related with transcriptional regulation may be involved in this process. Therefore, our microfluidic platform could become a functional assay for cells with different migratory to better identify key regulators for mechanistic studies and drug development.

## Conflict of Interest

The authors declare no conflict of interest.

## Supporting information

Supplementary

Supplementary

Supplementary

Supplementary

Supplementary
