# Supplementary material for: Single‐Cell Mobility Analysis of Metastatic Breast Cancer Cells
Source: Adv Sci (Weinh). 2018 Oct 31;5(12):1801158. doi: 10.1002/advs.201801158 (PMC6299679; doi:10.1002/advs.201801158)
Supplement: Supplementary file 1 — Supplementary [file ADVS-5-1801158-s002.pdf]

Copyright WILEY-VCH Verlag GmbH & Co. KGaA, 69469 Weinheim, Germany, 2018.

ADVANCED SCIENCE Open Access

Supporting Information

for *Adv. Sci.,* DOI: 10.1002/advs.201801158

Single-Cell Mobility Analysis of Metastatic Breast Cancer Cells

*Jialang Zhuang, Yongjian Wu, Liang Chen, Siping Liang, Minhao Wu, Ledu Zhou, Chunhai Fan, and Yuanqing Zhang**

Supplementary information

Single-cell mobility analysis of metastatic breast cancer cells

Jialang Zhuang[1+], Yongjian Wu[2+], Liang Chen[1], Siping Liang[2], Minhao Wu[2], Ledu Zhou[3], Chunhai Fan[4] and Yuanqing Zhang[1*]

[1]School of Pharmaceutical Sciences, Sun Yat-sen University, Guangzhou 510006, P. R. China;

[2]Department of Immunology, Zhongshan School of Medicine, Sun Yat-sen University, 74 Zhongshan 2nd Road, Guangzhou 510080, P. R. China;

[3]Department of General Surgery, Xiangya Hospital, Central South University, Changsha, Hunan 410008, P. R. China;

[4]Laboratory of Physical Biology, Shanghai Institute of Applied Physics, Chinese Academy of Sciences, Shanghai 201800, P. R. China;

+Both of these authors contributed to this study equally.

include:
Experimental Section
Supplementary Figures. S1–25
Supplementary Table S1-4
Captions for Videos S1, S2 and S3
Other Supplementary Materials for this manuscript includes:
Videos S1, S2 and S3
Supplementary Table S3

Experimental Section

Cell culture

MDA-MB-231 and MCF-7 cell lines were obtained from the American Type Culture Collection (ATCC; Manassas, VA). The SUM-159 cell line was obtained from Asterand (Detroit, MI). Lentiviral vector carrying GFP gene has been successfully constructed and maintains high expression in MCF-7, MDA-MB-231 and SUM-159 cells. These cells were grown in DMEM (Gibico), which was supplemented with 10% (vol/vol) FBS (Gibico), 1% penicillin-streptomycin, and 2 μg/mL blasticidin at 37° C in a humidified atmosphere of 5% CO_2. Cells were harvested by treatment with 0.25% trypsin-EDTA (Gibico). The MCPIP1 overexpression vector was cloned by inserting into the pcDNA3 control vector between Hind III and EcoR I sites. Briefly, for transient transfection, cells were seeded in six-well plates at a density of 4 × 10^5 cells/well. The following day, cells were transfected with 1000 ng/well of MCPIP1 expression vector using

Lipofectamine 3000 (Thermo Fisher Scientific) for 6 h. Following transfection, cells were maintained in medium plus 10% FBS for the times indicated in Results. The transfected cells were subjected to test cell mobility on chip or bulk RNA-sequencing.

Device design and fabrication.

Microstructure patterns were first designed in AutoCAD software and fabricated using standard photolithography and molding processes, as shown in Fig. S1. The printed out as 2-μm–resolution chrome masks were produced by Jixian optoelectronic Inc. (Shenzhen, China). The Silicon wafers (4-inch) were prepared by spin-coating the SU-8 3025 (MicroChem Corp) negative photoresist onto a silicon wafer and crosslinking via ultraviolet light for 7 s. Subsequently, the designed pattern was developed using SU-8 developer (MicroChem Corp.) and cleaned with isopropyl alcohol and nitrogen gas. The silicon masters were baked at 150°C for 30 min and then treated with the anti-adhesive agent TMCS (Sigma-Aldrich) via vapor reaction for 4 h.

Polydimethylsiloxane (PDMS RTV615) was obtained from Momentive Performance Materials (Waterford, NY). Next, the structure on the silicon wafer was used to fabricate the PDMS layer. The mold and PDMS layers were baked at 80°C for 2 h, and the cured PDMS was cut and removed from the mold. The holes for the inlets and outlets were punched using needle sizes that were compatible with the size of the fluid input/output pins. The PDMS layer was then cleaned by briefly rinsing with isopropyl alcohol and deionized water and dried with nitrogen gas. After treatment with oxygen plasma, the PDMS layer was bonded immediately to a glass slide. Finally, the bonded device was baked for 2 h at 80°C.

Cell loading

Cells were harvested by treatment with 0.25% trypsin-EDTA. After centrifugation, cells were resuspended in serum-free medium at a final concentration of 5×10^6 cells/ml. First, cells were loaded in the device from the main inlet by the application of pressure via a Laboratorial Syringe Pump (LongerPump, TS-2A). The flow is directed from the inlet to the outlets through 3 branches of main channels. The cell flow rate was maintained as 2.5 μL/min and about 12.5 μL or less of the cell suspension was gently introduced into the chip to seed cells in close proximity to the capture units within different microchannels. Second, after most of single cells were captured by the hooks, low serum culture medium (1%) were introduced into the inlet at a flow rate of 20 μL/min for 1 min in order to wash the uncaptured cells within the channels. Meanwhile, the residual cells in the main inlet were cleared up with micro pipette tips and washed with cell-free medium for three times.

Single cell mobility array

After cell loading, the chip will be settled in the microscope incubator (Tokai Hit). During the experiment, the temperature of the cell culture chamber was maintained at 37° C in a humidified atmosphere of 5% CO_2. Under this condition, single cell with different mobility will start migration after accommodation to microenvironment change. After 18 hours of cell migration, cultivation observations of the whole chip were collected for the profile of single cell migration.

Whole Chip Imaging

Imaging was performed using a high-resolution camera connected to an inverted optical microscope (Olympus, X81) equipped with objectives of 10× and 20×magnification. It cost about 5 minutes for taking the image of the whole chip (10×, bright field and FITC). Time-lapse images of the cell migration process were capture during the whole experiment. The migrated cells within the microchannel were identified at once and the cell migration paths were plotted (Figure S3). Migration distance of individual cells were quantified based on the on the distance form star line to the final cell frontier of each migration channels after 18 hours of incubation without medium replacement. After analysis of the raw data of the cell migrating distance, we can access the two key factors associated with cell mobility, that is, the migration distance of individual cells and a percentage of the high-migratory cells.

Cell Retrieval, single RNA-sequencing analysis and bulk RNA-sequencing analysis

First 20 μL of PBS was introduced into the inlet for 2 mintues for wash the whole microfluidic channels, followed by flowing 50 μL of trypsin in the inlet for 5 mintues in the stage top incubator. Then all single cells will flow toward different outlets and cell recovery was processed with microinjector. Single cells with different mobility were collected from individual outlets based on the migration profiles. According to cell mobility, cells were divided into two populations, high mobility cells and low mobility cells. Consequently, both of them were counted as three repicates of an amount of about 20 cells for single-cell RNA-sequencing. The single-cell suspensions were directly lysed for RNA purification, followed by the generation of cDNA through Smart-seq2 method for transcriptome sequencing. Briefly, these cells were transefered into lyasate buffer. Terminal deoxynucleotidyl transferase was used to add a poly(A) tail to the 3′ end of the first-strand cDNA, and then perform 21 PCR cycles to pre-amplify the single-cell cDNA. After cDNA purification by DNA clean beads, cDNA was resuspended in elution buffer (EB) (Qiagen). The fragmented cDNA was end-repaired, dA-tailed, adaptor ligated and then subjected to 10 cycles of PCR amplification. After cDNA amplification, libraries were quantified using Qubit 3.0 (Life Technologies) and quality of libraries was checked using Bioanalyser (Agilent 2100 Bioanalyzer). Using the Illumina HiSeq2000 sequencer, we generated 48 Gb of sequencing data from the 120 single cells, with, on average, 0.4 million reads per cell with read length of 100 bp.

For bulk RNA sequencing analysis, total RNA samples were extracted from cells between MDA-MB-231/Vector and MDA-MB-231/MCPIP1 cells, followed by the generation of cDNA through Smart-seq2 method for transcriptome sequencing. Briefly, after PBS washes, these cells were transefer into lyasate buffer. Terminal deoxynucleotidyl transferase was used to add a poly(A) tail to the 3′ end of the first-strand cDNA, and then perform 21 PCR cycles to pre-amplify the single-cell cDNA. After cDNA purification by DNA clean beads, cDNA was resuspended in elution buffer (EB) (Qiagen). The fragmented cDNA was end-repaired, dA-tailed, adaptor ligated and then subjected to 10 cycles of PCR amplification. After cDNA amplification, libraries were quantified using Qubit 3.0 (Life Technologies) and quality of libraries was checked using Bioanalyser (Agilent 2100 Bioanalyzer). Using the Illumina HiSeq2000 sequencer, we generated 98 Gb of sequencing data from 6 samples with read length of 150 bp.

Quality control was conducted: 1. The reads that aligned to adaptors with no more than two

mismatches. 2. The reads with more than 10% unknown bases (N bases). 3. The reads with more than 50% of low-quality bases (quality value ≤ 5) in one read. We subsequently only used samples with Q20 > 75% and a rate of clean data (percentage of obtained final data with respect to raw data) of >45% for further analysis.

After the RNA library preparation, reads were aligning to GRCh38 (hg19 RefSeq), which as downloaded from the UCSC Genome Browser (http://genome.ucsc.edu), with HISAT2. Based on the above results, gene expression levels were estimated by StringTie. FPKM (expected number of Fragments Per Kilobase of transcript sequence per Millions base pairs sequenced) (calculated using Cufflinks (v 2.2.1)) was used to access the gene expression levels. Differential expression between the putative groups was conducted using the R package DESeq2, genes which were expressed at least 5 read counts in 3 samples per group would take into consideration. PCA analysis for single-cell RNA-seq was performed by R package ggplot based on all detected gene. We identified genes with significant differences in expression between high migratory and low migratory cells as defined by P-values < 0.05 and absolute values of the logarithm (to basis 2) of the fold change (Log_2FC) > 1. HemI was used for visualization of gene expression signature. Functional enrichment analysis based on gene ontology (GO) database was performed by using David 6.7 program (http://david.abcc.ncifcrf.gov/). The enriched biological processes with adjusted P value < 0.01 (Fisher's exact test) were selected and shown in the corresponding Supplementary Figures.

RNA extraction and real-time PCR.

Total RNA was extracted from cells with TRIzol reagent (Invitrogen Life Technologies, Inc., Carlsbad, CA) and quantified by NanoDrop 2000 spectrophotometer. 1 μg of total RNA was reversely transcribed to produce cDNA by using RevertAid First Strand cDNA synthesis kit (Thermo Fisher Scientific, Waltham, MA, USA), and then amplified using SYBR Green Master Mix (Thermo Fisher Scientific, Waltham, MA, USA) following the manufacturer's protocol. Quantitative real-time PCR reactions were performed using the CFX96 Real-Time PCR System (Bio-Rad, Hercules, CA). The average threshold cycle (CT) values of samples were normalized to CT of β-actin gene. The relative expression was determined by the $2^{-\Delta\Delta}$ CT method. The relevant primers were as follows: MCPIP1 primers 5' -GCTGCTCATGTACTCCTTCGTC-3' and 5'-CAAAGTGAGTGGCTTCTTACGC-3', and β-actin primers 5'-GAGCACAGAGCCTCGCCTTT-3' and 5'-ACATGCCGGAGCCGTTGTC-3'.

Western blot analysis.

Cells were washed twice with PBS containing 1 mM PMSDF and RIPA Lysis Buffer (Beyotime). Then, protein concentration of the supernatant was determined by Quick Start Bradford protein assay (Bio-Rad). 20 μg of each sample was loaded, separated on 12% SDS-PAGE, and then transferred to a supported nitrocellulose membrane (Pall Life Sciences, Ann Arbor, MI). After blockage, the membranes were incubated overnight in TBST/5% milk containing anti-MCPIP1 (1:1000, Clone ab97910, Abcam,) or anti-β-actin (1:1000, Clone ab8227, Abcam) antibodies at 4°C, followed by incubation with appropriate HRP-conjugated secondary antibodies at room temperature for 1 hour. Finally, the membranes were visualized with New-SUPER ECL (KeyGEN, Nanjing, China) according to the manufacturer's protocol.

Establish of Pulmonary Metastasis Model

BALB/c nude mice were purchased from Laboratory Animal Center of Sun Yat-sen University (Guangzhou, GD, CN). The mice (N = 5 per treatment group) were intravenously injected with 1×10^6 MDA-MB-231 or MCF-7 cells (200 μl volume injections), and allowed to establish metastatic tumors primarily in lungs. Mice were divided into four groups, including transference with (1) MCF-7, (2) MDA-MB-231/Vector, (3) MDA-MB-231/MCPIP1 (4) MDA-MB-231/Vector, pretreated with SB431542 (10 μM, Selleckchem). After 1 month, lungs from mice were weighed and photographed. Lungs were sliced and stained with hematoxylin-eosin (H&E).

Wound healing assays

Cells were plated in 96-well plates in triplicate. After 24 hours, the cells were treated overnight with mitomycin C (5 μg/mL, Santa Cruz Biotechnology). Then, a wound was made in the monolayer with a pipette tip, the medium was replaced by serum-free medium. Pictures of the wounds were taken 0 and 18 hours after treatment initiation, and wound closure was measured using ImageJ software.

Transwell Migration assay

Transwell migration assays were performed using a 24-well Transwell chamber system (Costar 3422, Corning Inc., NY, USA). Cells were seeded in the upper chamber in 0.1 ml serum-free DMEM media. Media supplemented with 10% fetal bovine serum was placed in the bottom well in a volume of 0.8 ml (used as a chemoattractant). After incubation for 18 h, migrated cells on the lower surface were stained with crystal violet stain and counted under a light microscope.

Statistical analysis

SPSS 20.0 was used to test for the normality of data of single cell migration distances, which were performed by Kolmogorov-Smirnov Test and the Shapiro-Wilk Test. Descriptive Statistics and the histogram for the frequency distribution of data of single cell migration distances were performed by SPSS 20.0. For comparing the mobility among different groups of single cells, analysis based on Mann-Whitney U test performed as a nonparametric test for cell migration distances was performed using GraphPad Prism 6. For comparing the numbers of nodule and weight of lung among different treated-groups *in vivo*, Student's t-test (one-tailed) was used by GraphPad Prism 6.

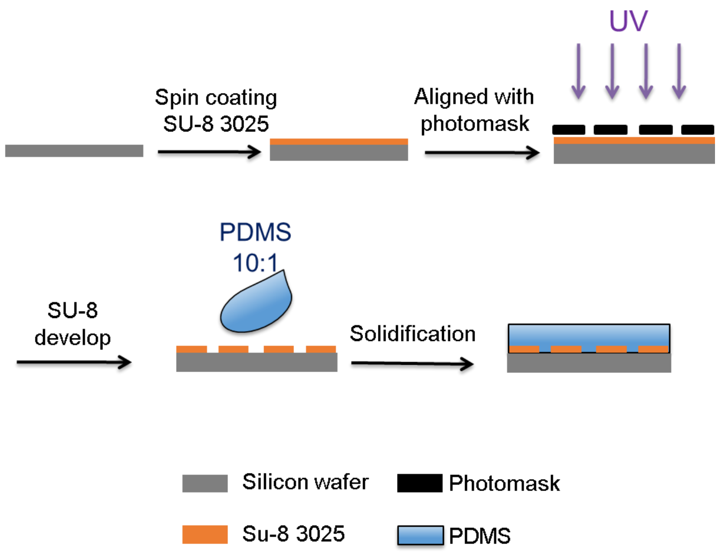

Figure S1. Schematic representation of the photolithography procedures.

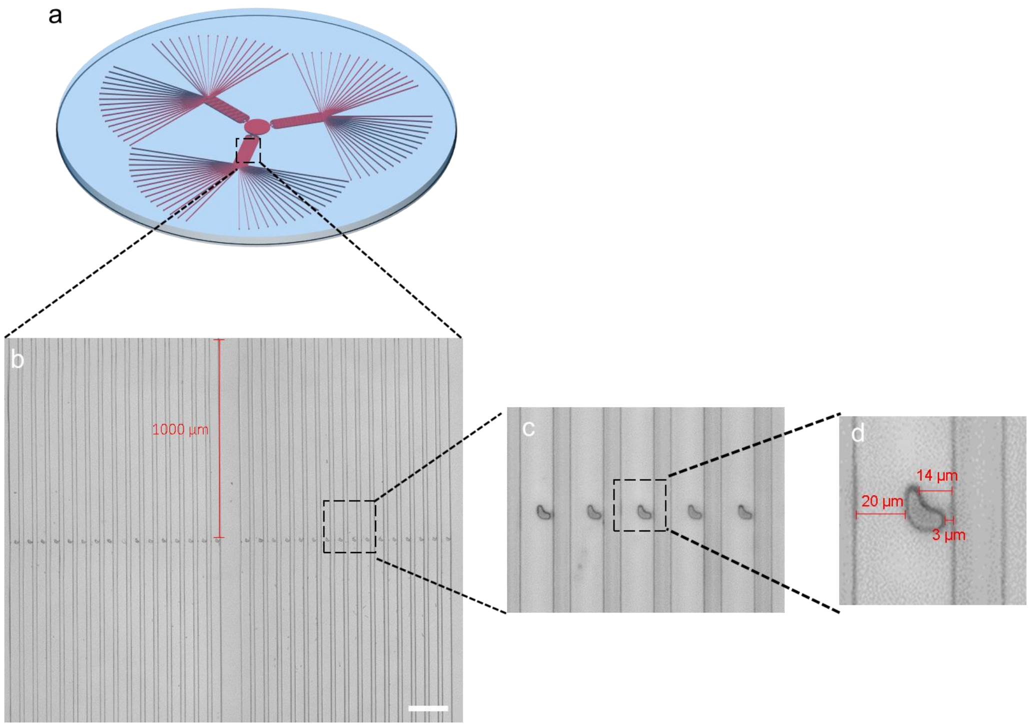

Figure S2. Optical micrographs of the microchannel and microscale hook in the SCM-Chip. a) Schematic representation of the SCM-Chip, scale bar, 200 μm. b) One of three multi-microchannel branches in SCM-Chip, there are 32 microchannels in one branch of the SCM-Chip. c) All the hooks were placed in the same level within a branch. d) The lowest gap and the largest gap between the hook and microchannel are 3 μm and 14 μm, respectively.

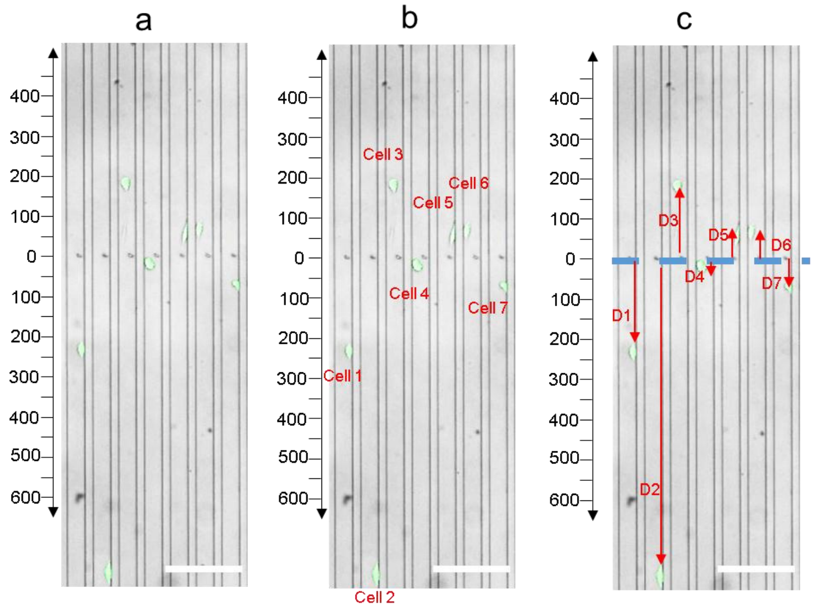

d

Move path	Distance (μm)
D1	157
D2	430
D3	105
D4	20
D5	53
D6	51
D7	65

Figure S3. Measure procedure of the Single-cell migration after 18 hours movement.

a) Raw capture image of the whole chip after 18 hours migration. b) Identify the migrated cells within the microchannel. c) Calculate the distance of cells from the start line, scale bars, 200 μm. d) data of the Single-cell migration calculated based on c).

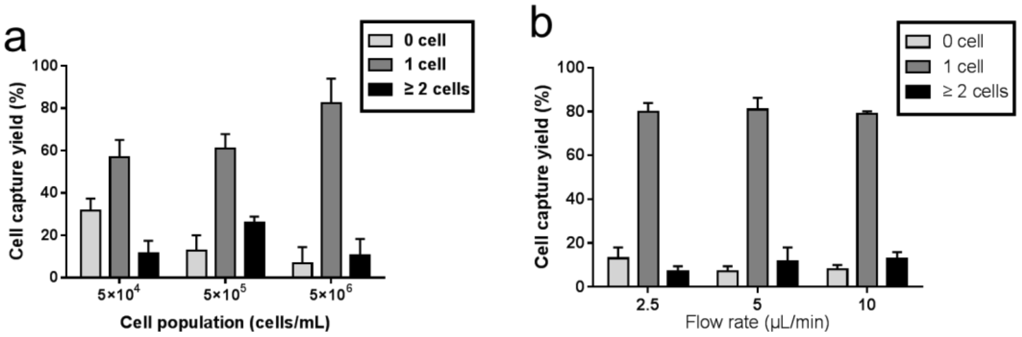

Figure S4. Optimization and of SCM-Chip for single-cell capture.

Single-cell capture efficiency under various cell population a) and flow rate b).

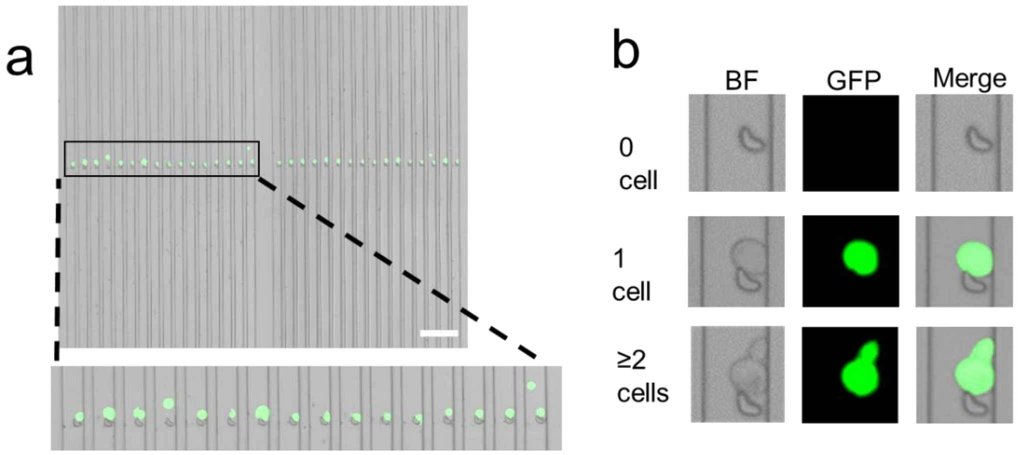

Figure S5. Characterization of SCM-Chip for single-cell capture.

a) Uniform Single-cell loading at the microscale hooks of different migration channels, scale bar,

200 μm. b) Fluorescence images of Single-cell capture. Up, hook with no cell, middle, hook with one Single-cell, low, hook with more than one Single-cell.

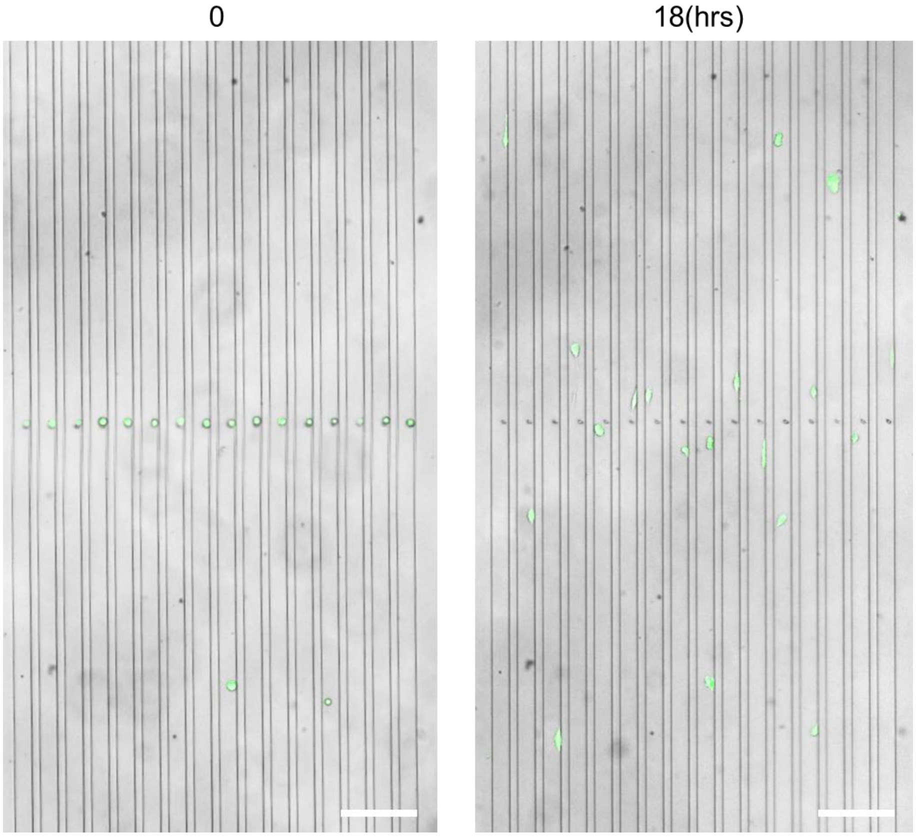

Figure S6. Single cell migration of MDA-MB-231 on chip.

Overlay Images of bright field and GFP channel of Single-cell migration of MDA-MB-231 cells before and after 18 hours movement, scale bars, 200 μm.

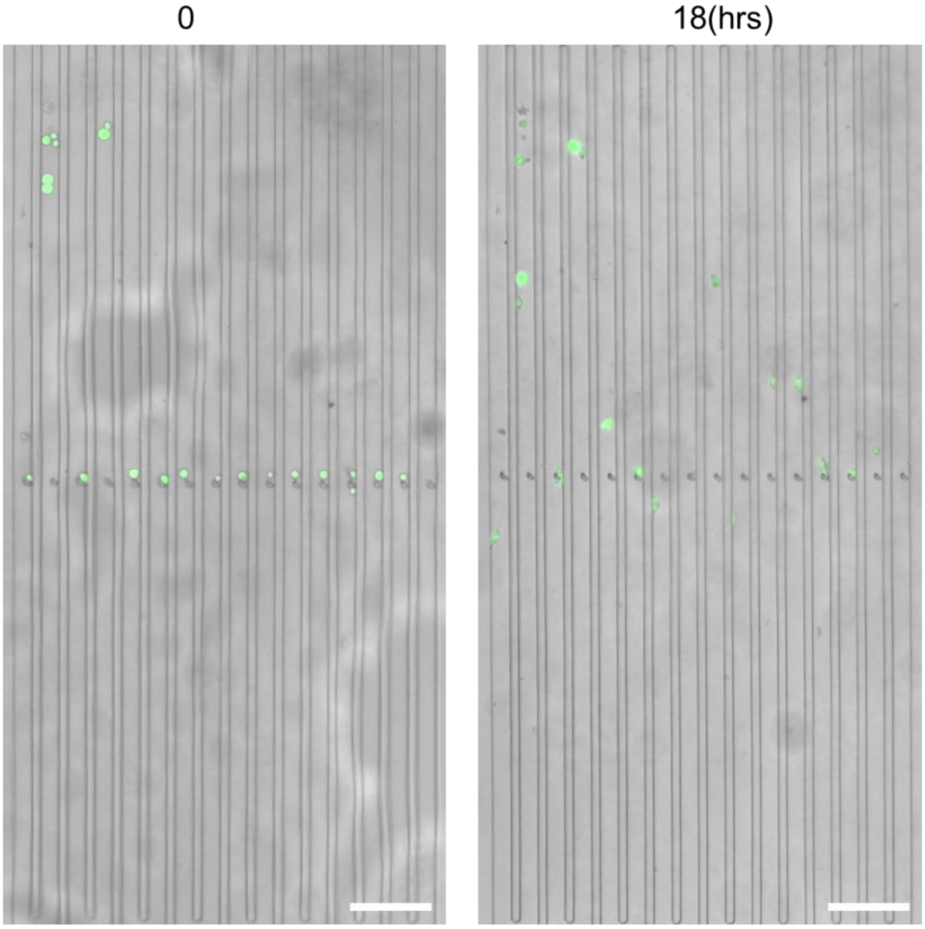

Figure S7. Single cell migration of MCF-7 on chip.

Overlay Images of bright field and GFP channel of Single-cell migration of MCF-7 cells before and after 18 hours movement, scale bars, 200 μm.

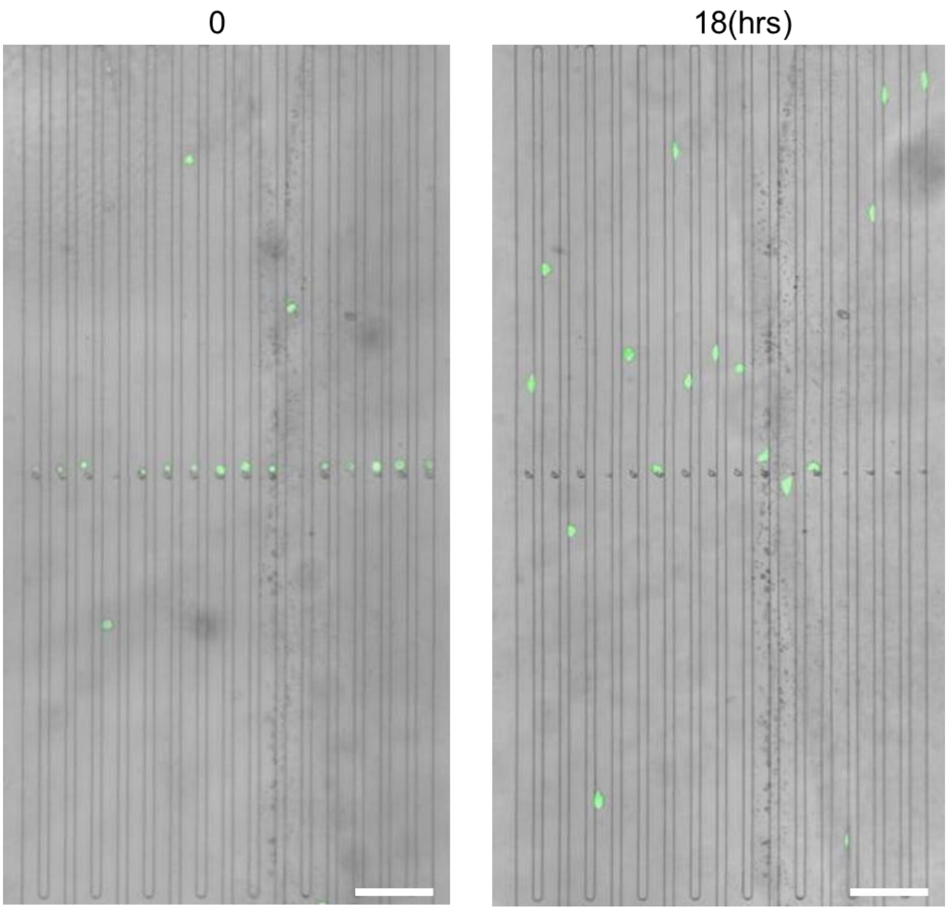

Figure S8. Single-cell migration of SUM-159 on chip.

Overlay Images of bright field and GFP channel of Single-cell migration of SUM-159 cells before and after 18 hours movement, scale bars, 200 μm.

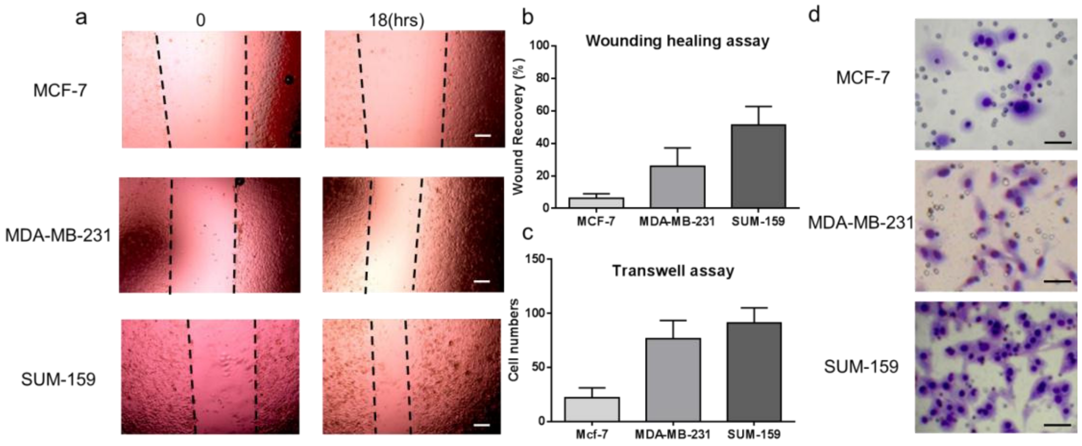

Figure S9. Single-cell mobility array exhibit similar results with that performed by traditional migration assay.

Microscopic images a) and healing rates b) of scratch wound healing assay among three breast cancer cell lines, scale bars, 200 μm. Relative migration rates c) and microscopic images d) of transwell migration assay among three breast cancer cell lines, scale bars, 20 μm.

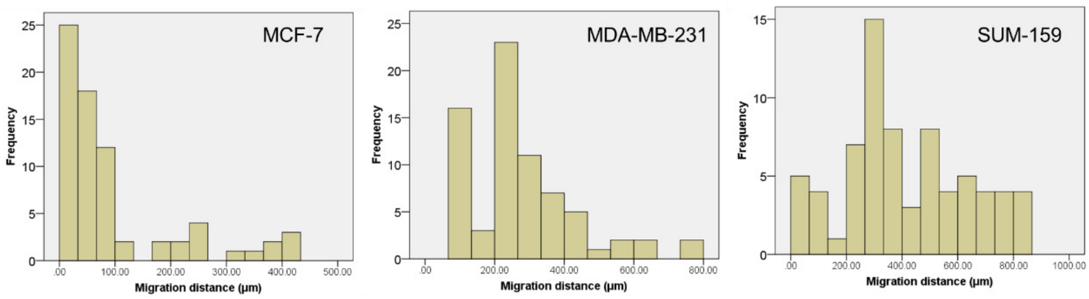

Figure S10. Histogram for the frequency distribution of cell migration distance among three breast cancer cell lines

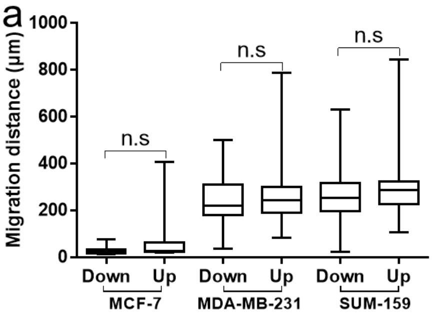

b

Cell line	No-migrated	Migrating Down	Migrating Up	P value
MCF-7	65	3	3	0.6433
MDA-MB-231	49	20	15	0.0886
SUM-159	22	24	31	0.2770

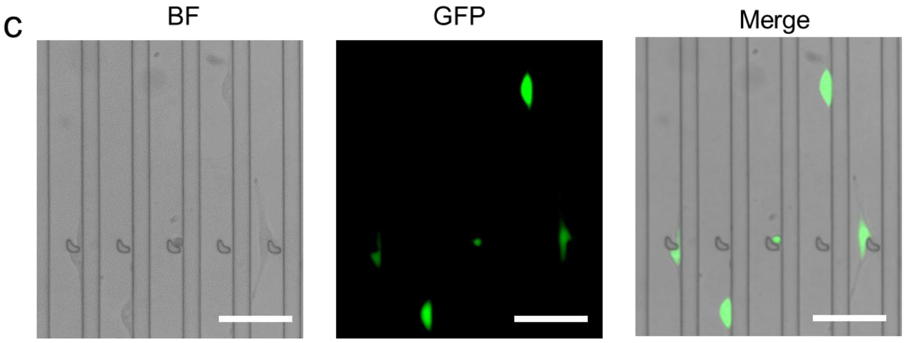

Figure S11. No difference between up-migration and down-migration among three breast cancer cell lines.

The migration distances a) and comparison of the amount b) between up-migration and down-migration cells among three breast cancer cell lines. c) Fluorescence images of migrating cells within migration channel in SCM-Chip. There are up-migration and down-migration cells during the migration process, scale bars, 100 μm.

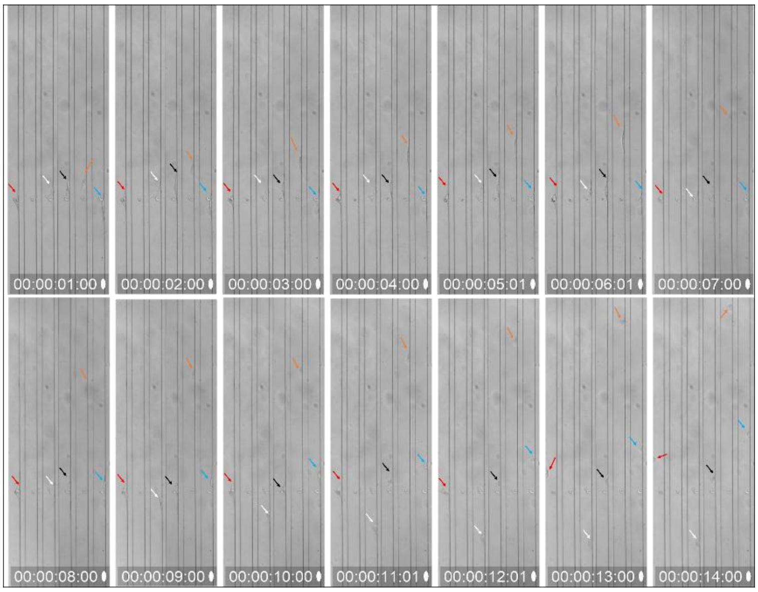

Figure S12. Real-time monitoring of MDA-MB-231 cells within the SCM-chip during 14 hours migration. Arrows with different colors indicate different migrating cells.

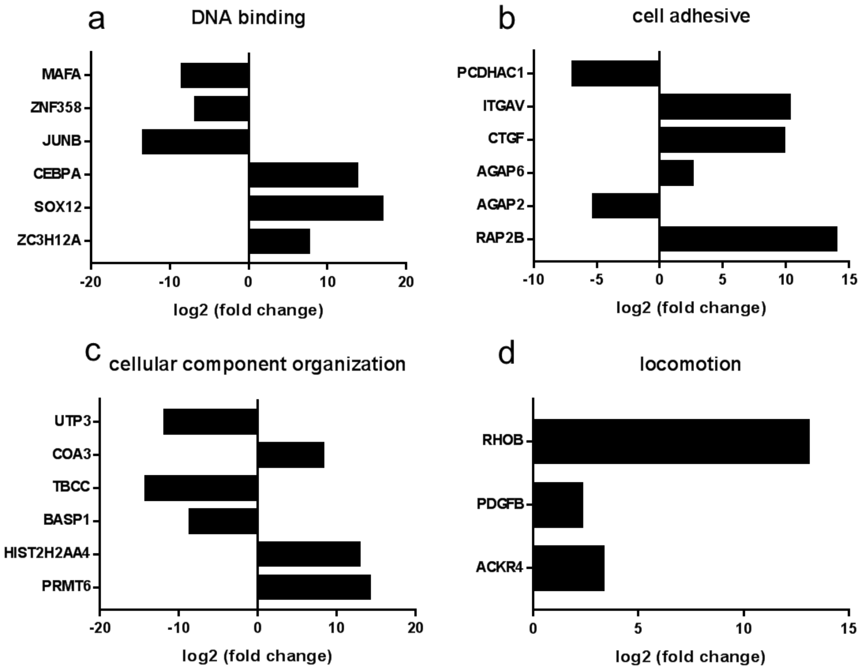

Figure S13. Fold change of gene expressions between high-migratory cells and low-migratory cells sorted from SCM-chip.

Relative major differences in the expression of DNA binding a), Cell adhesive b), Cellular component organization c) and Locomotion d) genes in high mobility cells compared to low mobility cells during single-cell migration

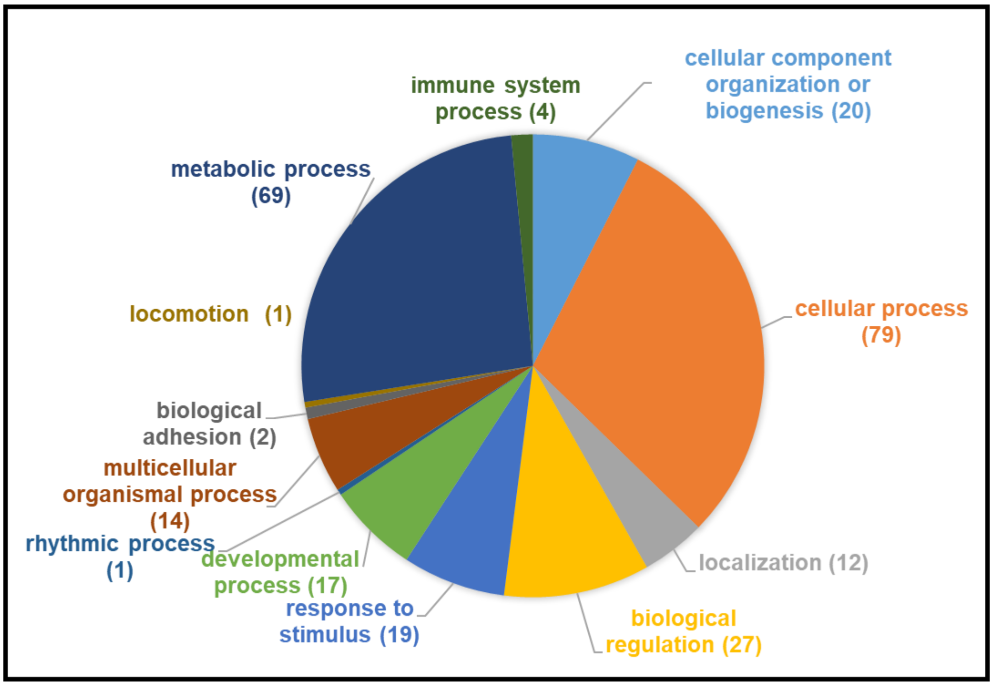

Figure S14. Pie chart of the 12 most significant enrichment terms of differently expressed genes across these two subpopulations

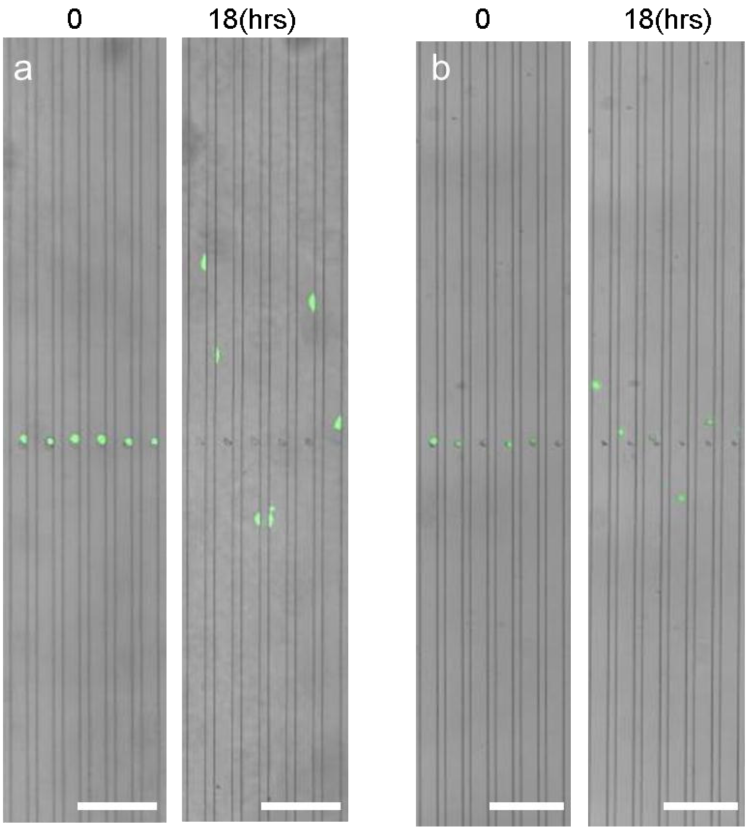

Figure S15. Over expression of MCPIP1 can decrease single-cell migration.

Overlay Images of bright field and GFP channel of Single-cell migration of MDA-MB-231/Vector

a) and MDA-MB-231/MCPIP1 b) before and after 18 hours movement, scale bars, 200 μm.

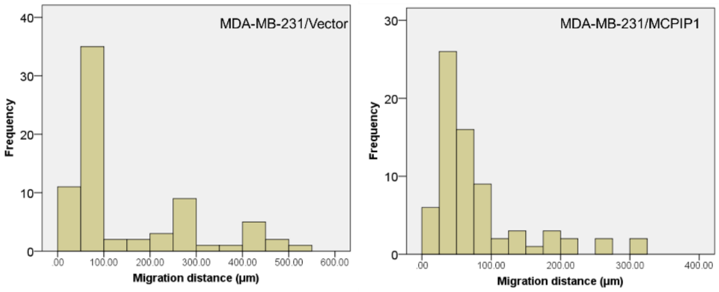

Figure S16. Histogram for the frequency distribution of cell migration between MDA-MB-231/Vector and MDA-MB-231/MCPIP1 cells

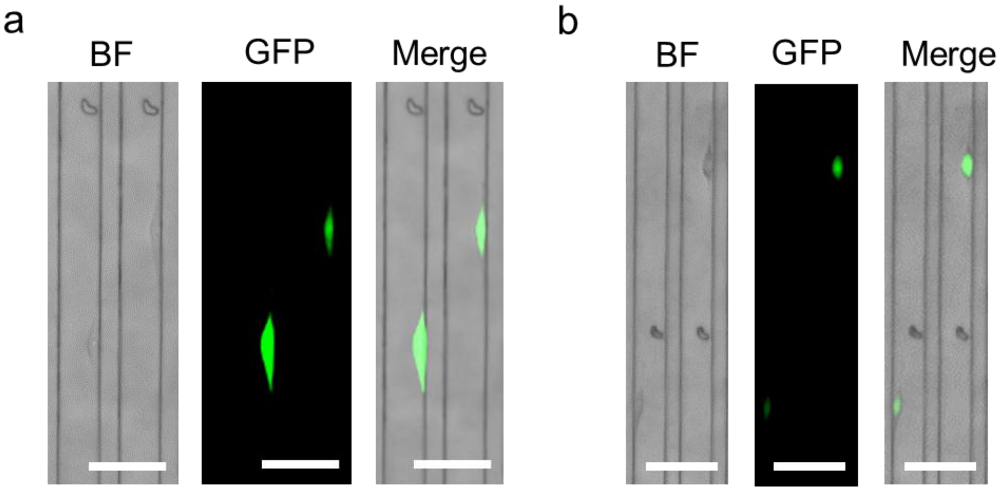

Figure S17. Over expression of MCPIP1 change the morphologies of single-cell migration on chip.

Comparison of the morphologies of Single-cell migration between MDA-MB-231/Vector a) and MDA-MB-231/MCPIP1 b), scale bars, 100 μm.

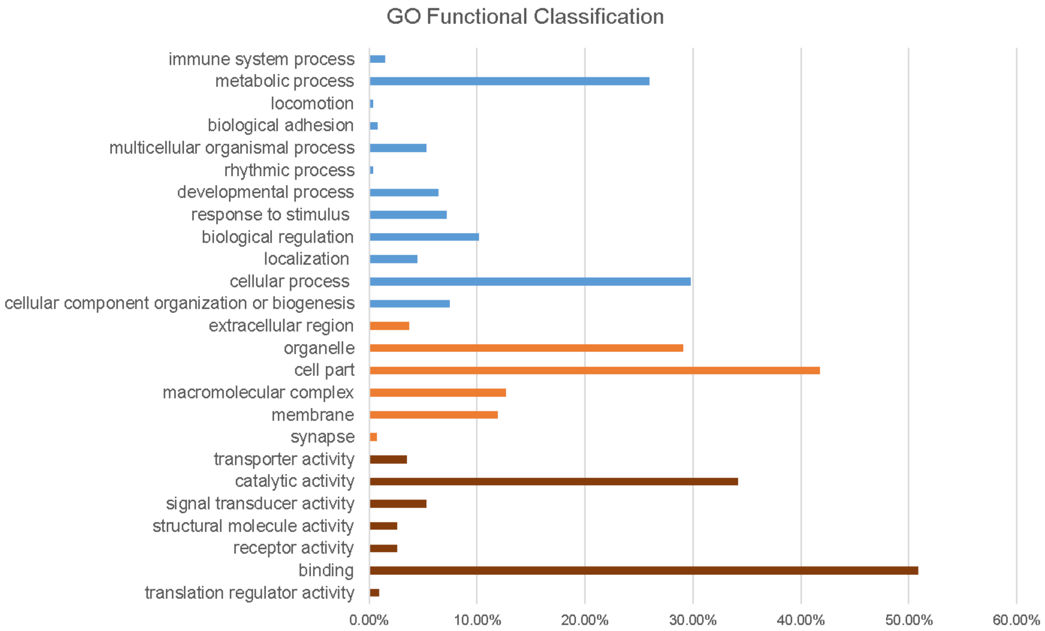

Figure S18. Go functional classification of differentially expressed genes between cells with different expressions of MCPIP1.

The most enriched gene ontology of differentially expressed genes of biological function (blue column), cellular component (orange column) and molecular function (brown column) ($p < 0.05$, false discovery rate < 0.05) between MDA-MB-231/Vector and MDA-MB-231/MCPIP1.

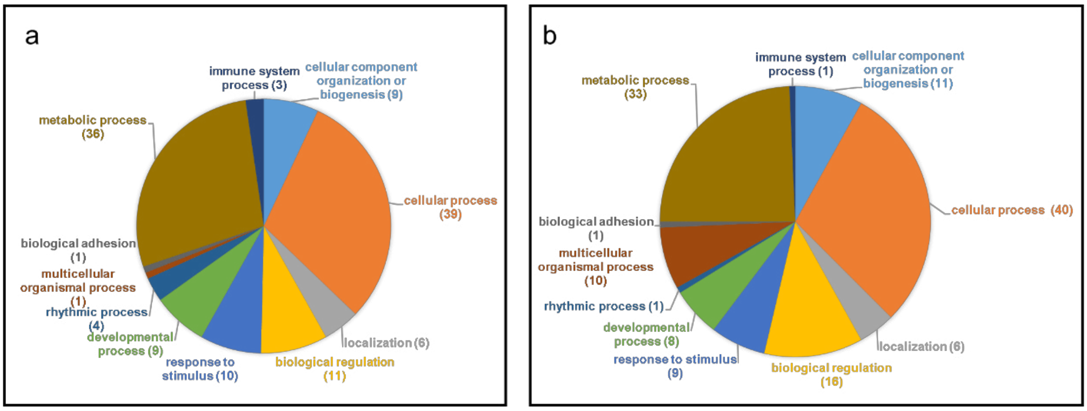

Figure S19. Gene set enrichment analysis between cells with different expressions of MCPIP1. The pie chart of the 11 most significant enrichment terms of over-expressed a) and down-expressed b) genes between MDA-MB-231/Vector and MDA-MB-231/MCPIP1.

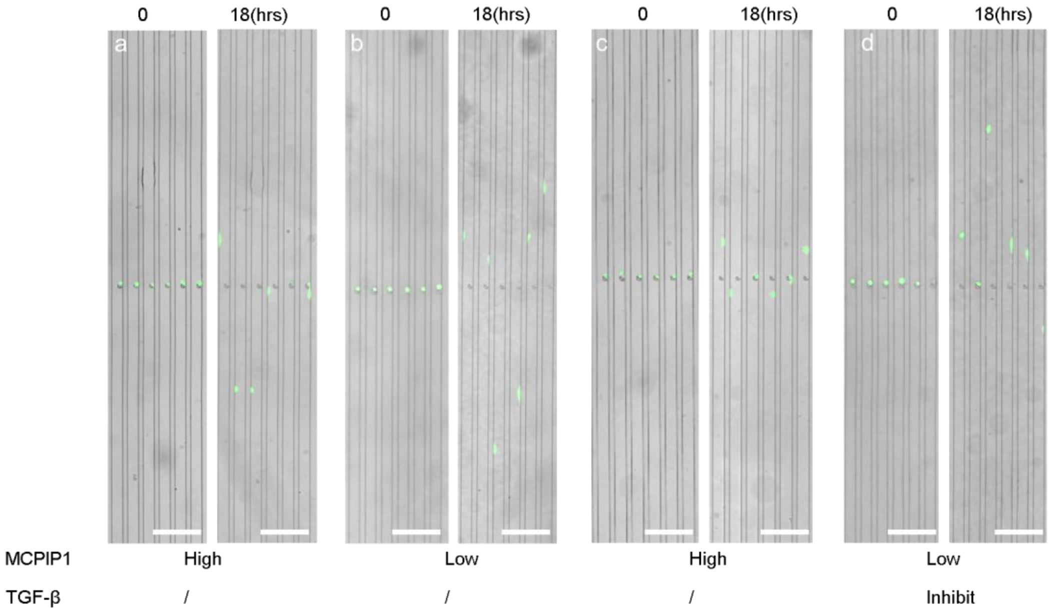

Figure S20. Inhibition of TGF-β pathway can decrease single-cell migration among low-MCPIP1 cells. Overlay Images of bright field and GFP channel of Single-cell migration of MCF-7/Vector a), MDA-MB-231/Vector b), MDA-MB-231/MCPIP1 c) and SB431542 (10 μM) pretreated MDA-MB-231/Vector cells d) before and after 18 hours movement, scale bars, 200 μm.

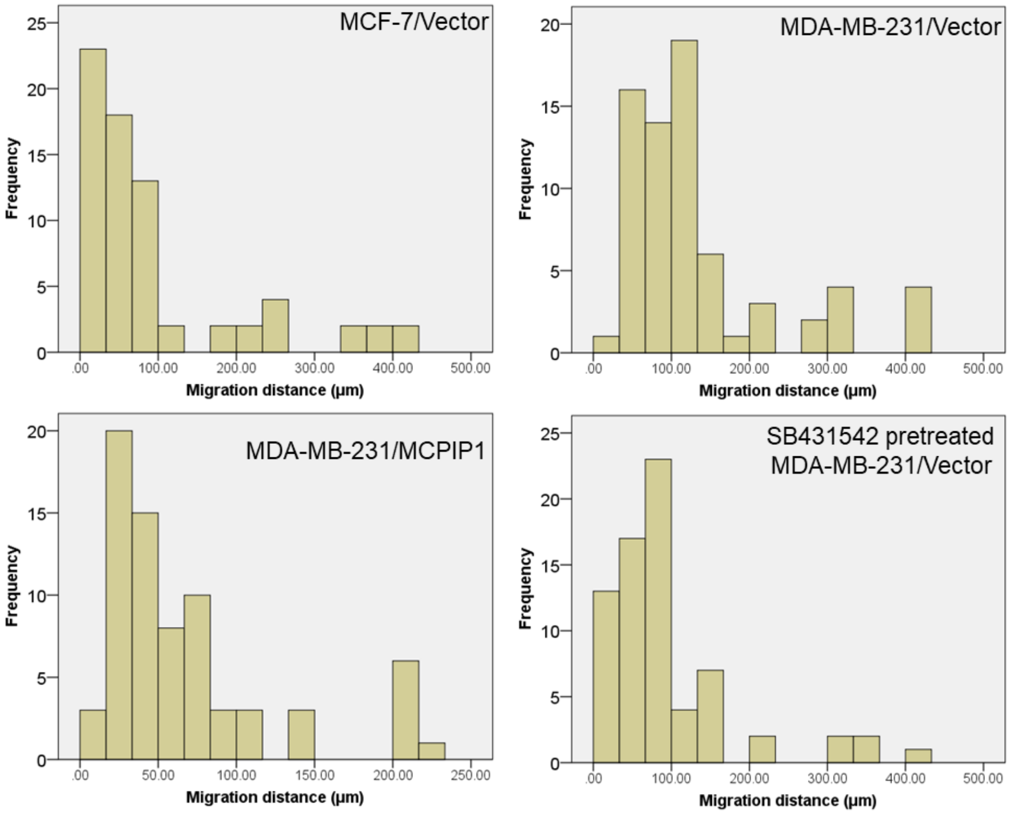

Figure S21. Histogram for the frequency distribution of cell migration of MCF-7/Vector, MDA-MB-231/Vector, MDA-MB-231/MCPIP1 and SB431542 (10 μM) pretreated MDA-MB-231/Vector cells.

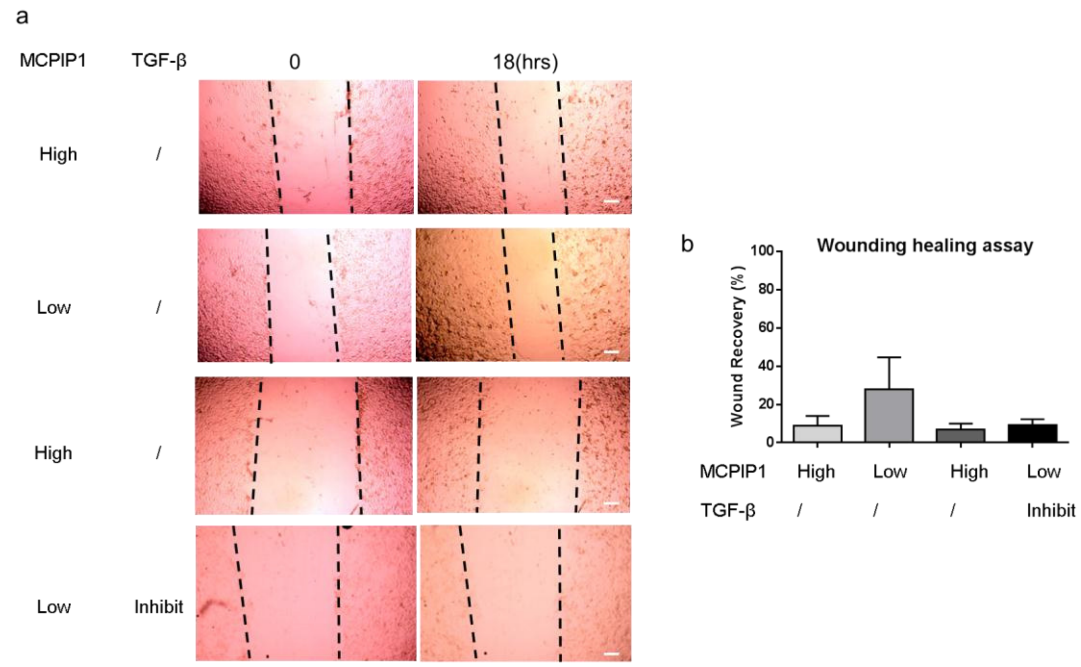

Figure S22. Inhibition of TGF-β pathway can prevent wound recovery of low-MCPIP1 cells. Microscopic images a) and healing rates b) of scratch wound healing assay among MCF-7/Vector, MDA-MB-231/Vector, MDA-MB-231/MCPIP1 and SB431542 (10 μM) pretreated MDA-MB-231/Vector cells, scale bars, 200 μm.

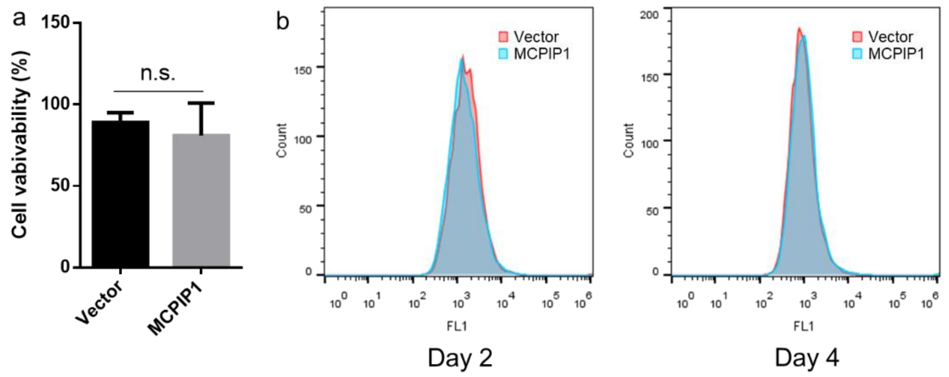

Figure S23. Overexpression of MCPIP1 in MDA-MB-231 cells did not affect their proliferation potential. a) Cell viability assay between MDA-MB-231/Vector and MDA-MB-231/MCPIP1 cells on day 1(mean ± SD, n = 5), b) CFSE profiles of MDA-MB-231 cells with different expression of MCPIP1 proliferation. CFSE profiles of cells that were analyzed on 2 day and 4 day after CFSE labeling. (cell count = 5000)

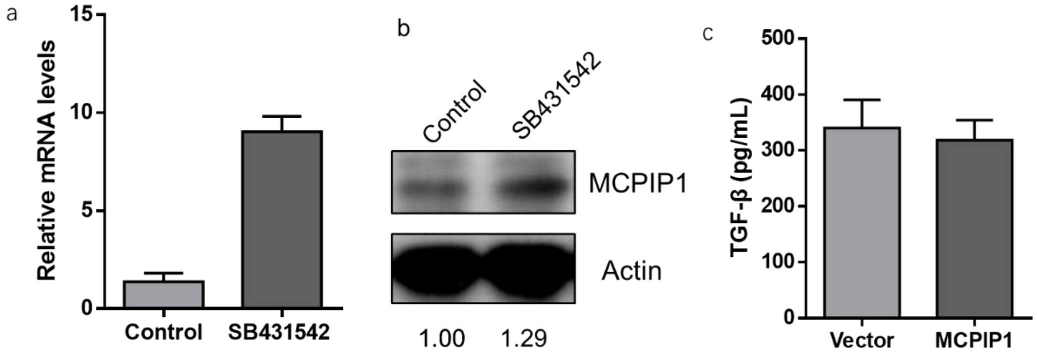

Figure S24. TGF-β inhibition results in increase of MCPIP1 and overexpression of MCPIP1 did not affect protein expression of TGF-β. a) qRT-PCR analysis of MCPIP1 mRNA level in MDA-MB-231, and SB431542 (10 μM) pretreated MDA-MB-231 cells (mean ± SD, n = 3). b) Western-blot analysis of MCPIP1 protein level in MDA-MB-231, and SB431542 (10 μM) pretreated MDA-MB-231 cells. c) total TGF-β expression of MDA-MB-231 cells with different expression of MCPIP1 (mean ± SD, n = 3)

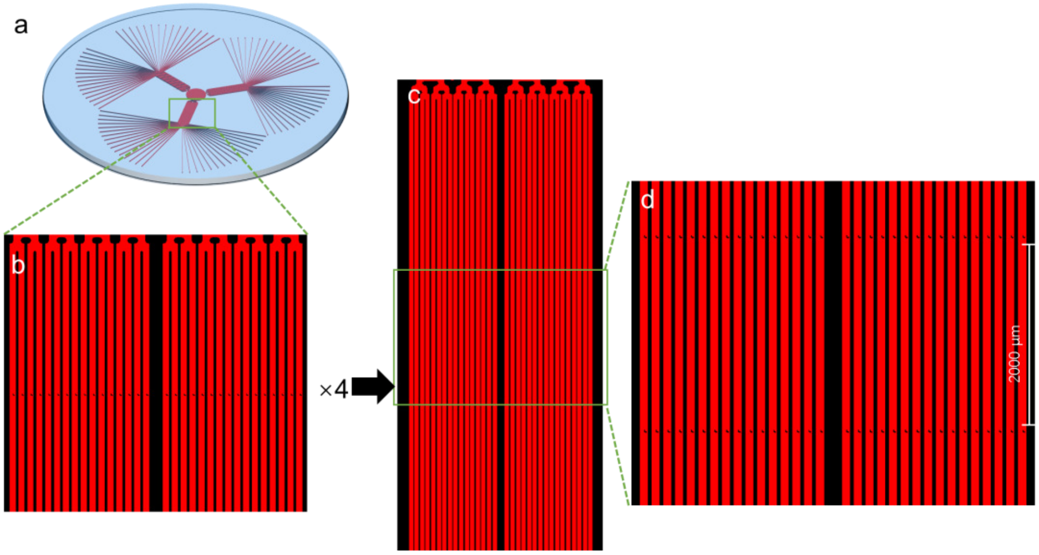

Figure. S25. Solution to higher throughput for Single-cell mobility study.

a) Schematic representation of the SCM-Chip, b) Auto CAD drawing of the multi-microchannel in SCM-Chip, Auto CAD drawing of the partial branch of multi-microchannel c) and the detail design (d) of 384-throghput SCM-Chip

Supplementary Table

Table S1. The calculated relative migration speed of three breast cancer cell lines based on the raw data from SCM-Chip.

Cell line	cell velocity (μm/min)
MCF-7	0.10 (0.01-0.43)
MDA-MB-231	0.21 (0.04-0.82)
SUM-159	0.40 (0.03-0.88)

Table S2. Descriptive Statistics of the migration distance between three breast cancer cell lines

Group	Sample size	Mean	Std. Error	95% Confidence Interval for Mean	
				Lower Bound	Upper Bound
MCF-7	71	95	18	58	132
MDA-MB-231	85	203	14	174	231
SUM-159	78	386	27	333	439

Table S3. Differentially expressed genes between high-migratory cells and low-migratory cells sorted from SCM-chip

See Extended file Table S3

Table S4. Descriptive Statistics of the migration distance between MDA-MB-231/Vector and MDA-MB-231/MCPIP1 cells

Group	Sample size	Mean	Std. Error	95% Confidence Interval for Mean	
				Lower Bound	Upper Bound
MDA-MB-231/Vector	80	186	20	146	227
MDA-MB-231/MCPIP1	69	75	10	56	95

Table S5. Descriptive Statistics of the migration distance of MCF-7/Vector, MDA-MB-231/Vector, MDA-MB-231/MCPIP1 and SB431542 pretreated MDA-MB-231/Vector cells

MCPIP1	TGF-β	Sample size	Mean	Std. Error	95% Confidence Interval for Mean	
					Lower Bound	Upper Bound
High	/	71	90	16	56	122
Low	/	73	172	19	131	212
High	/	75	69	8	53	86
Low	Inhibit	83	96	12	73	119

Supplementary Videos

Video S1. Single-cell capture on the chip. Cells suspension was introduced into the inlet with a flow rate of 2.5 μL /min. Most of Single-cells can be captured by the hooks, and the others flowed along the bypass channel.

Video S2. Single-cell retrieval on the chip. After 18 hours migration, trypsin were flowed into the inlet, and the single cells can be collected from different outlets.

Video S3. Single-cell migration on the chip. After 1 hour incubation, the chip was placed in the stage top incubator. Single-cells within isolated microchannel start moving along the PDMS wall and maintain persistent migration in the same direction. Time is shown in hour:min.
